# The effect of bispectral index monitoring on cognitive performance following sedation for outpatient colonoscopy: a randomized controlled trial

**DOI:** 10.1590/1516-3180.2018.0383210519

**Published:** 2019-09-09

**Authors:** Mehmet Sargin, Mehmet Selçuk Uluer, Barış Şimşek

**Affiliations:** I MD, Assistant Professor, Anesthesiology and Reanimation Department, Selçuk Üniversitesi Tıp Fakültesi, Konya, Turkey.; II MD. Physician, Anesthesiology and Reanimation Department, Health Sciences University, Konya Eğitim ve Araştırma Hastanesi, Konya, Turkey.; III MD. Physician, Anesthesiology and Reanimation Department, Health Sciences University, Konya Eğitim ve Araştırma Hastanesi, Konya, Turkey.

**Keywords:** Consciousness monitors, Cognition, Conscious sedation, Colonoscopy

## Abstract

**BACKGROUND:**

Bispectral index (BIS) monitoring can positively affect cognitive performance through decreasing the use of sedative agents. We aimed to evaluate the effect of BIS monitoring on early cognitive performance among patients undergoing sedation for colonoscopy.

**DESIGN AND SETTING:**

Randomized, controlled trial in a university hospital.

**METHODS:**

100 patients were randomized into two groups. In the monitored group (n = 50), the depth of anesthesia was monitored using the BIS, and BIS scores were maintained between 60 and 80. In the usual care group (n = 50), BIS monitoring was not performed. To determine the patients’ baseline cognitive performance levels, the mini-mental state examination (MMSE), Trieger dot test (TDT) and clock drawing test (CDT) were used. The patients’ post-procedure cognitive performance levels were determined when they were classified as ready for discharge.

**RESULTS:**

The total volume (mg) of propofol used [median (range) IQR] in the sedation procedure was lower in the monitored group [100 (50-200) 100-140] than in the usual care group [150 (75-200) 100-200] (P < 0.001). The discharge scores [mean (SD)] using MMSE and CDT were higher in the monitored group [26 (3) and 3 (1), respectively] than in the usual care group [23 (3) and 2 (1), respectively] (P = 0.002 and P = 0.002, respectively). The discharge scores using TDT [mean (SD)] were lower in the monitored group [11 (7)] than in the usual care group [15 (11)] (P = 0.033).

**CONCLUSION:**

BIS monitoring among sedated patients was associated with lower propofol use and smaller decline in cognitive performance.

**CLINICAL TRIAL REGISTRATION:**

This trial was registered in the Australian New Zealand Clinical Trial Registry (ACTRN12617000134325).

## INTRODUCTION

Colonoscopy is one of the most commonly performed procedures worldwide. However, it frequently results in pain and discomfort in patients undergoing the procedure. Cooperation from the patient is crucial for obtaining clear endoscopic images. Effective sedation ensures that the patient can tolerate the procedure and cooperate well, in addition to facilitating recovery, all of which increase the satisfaction of both the patient and the endoscopist. Sedation has the aim of facilitating endoscopy, but it may also prolong hospitalization and result in cognitive impairments that can affect daily activities.^1^

Postoperative cognitive dysfunction (POCD) is a relatively common entity that can adversely affect recovery and discharge after outpatient procedures.^2^ A review of the literature has indicated that the incidence of POCD ranges from 7% to 60%, depending on the patient group and type of procedure.^3^ Postoperative cognitive problems can be evaluated in terms of several categories, and previous studies have suggested different diagnoses and classifications.^4^^,^^5^ POCD can be diagnosed based on the changes noted in psychomotor test scores, e.g. using the digit-symbol-substitution test (DSST), Trieger dot test (TDT) or mini-mental state examination (MMSE), in comparison with the pre-procedural values.^6^ POCD also affects a wide range of cognitive functions, including memory, attention, orientation and concentration, and these effects may persist for months after operations, in certain patients.^7^

The mechanism underlying POCD is still unclear. It is a challenge to establish a definitive conclusion regarding the mechanism underlying this condition, given the differences between patient populations, the tools used for diagnosing it and the variations between analyses of cognitive test results in the literature.^4^ However, old age, low levels of education and low preoperative cognitive reserves have been implicated as risk factors for the development of postoperative cognitive dysfunction.^4^^,^^5^^,^^8^^,^^9^

A bispectral index (BIS) monitor is commonly used to assess depth of sedation when administering sedative, hypnotic or anesthetic agents during surgical and medical procedures.^10^ Monitoring the level of sedation by means of safe objective methods such as BIS monitoring makes it possible to use lower amounts of sedative agents.^11^ Previous studies on the use of BIS for sedation monitoring during colonoscopy mostly focused on decreasing the amount of sedative agent administered and the recovery time required, and on assessing the patient’s or endoscopist’s satisfaction.^12^^,^^13^^,^^14^^,^^15^^,^^16^

The influence of depth of anesthesia on postoperative impairment of cognitive function among surgical patients has already been investigated through BIS monitoring.^17^ Various medications have been compared in studies assessing postoperative impairment of cognitive function subsequent to procedures such as colonoscopy and endoscopic retrograde cholangiopancreatography (ERCP).^1^^,^^18^ However, no previous study has evaluated the effects of BIS monitoring on early cognitive performance after outpatient procedures such as colonoscopy and ERCP.

The reduction in consumption of anesthetic agent achieved through BIS monitoring has been shown to result in improvement in the early recovery profile.^19^^,^^20^^,^^21^ However, it is still unclear whether the lower dose of anesthetic agents administered with the aid of BIS monitoring reduces the risk of postoperative cognitive dysfunction.

## OBJECTIVE

We conducted a randomized controlled trial to evaluate the effect of BIS monitoring on early cognitive performance among patients undergoing sedation for colonoscopy. The primary aim was to evaluate cognitive performance and the secondary aims were to evaluate the effect of BIS monitoring on total propofol use, duration of sedation and patient satisfaction.

## METHODS

### Ethics

We declare that implementation of this study was endorsed by the Internal Review Board (Ethics Committee) of Meram School of Medicine, Necmettin Erbakan University, under the date and approval number 04/12/2015/2015/366. Furthermore, informed written consent was obtained from all patients between January 30, 2017, and January 15, 2018. This study was registered with the Australian New Zealand Clinical Trial Registry (ACTRN12617000134325).

### Trial design and setting

This was a parallel, randomized controlled trial conducted at Konya Training and Research Hospital, Health Sciences University, Konya, Turkey.

### Participants

Patients between the ages of 18 and 70 years, who presented American Society of Anesthesiologists (ASA) physical status I-III and had been scheduled to undergo planned colonoscopy, were studied.

Patients with inadequate comprehension of Turkish, mini-mental state examination score ≤ 23, significant cardiorespiratory instability (ASA IV-V), prior administration of intravenous fluid, allergies to eggs, beans or latex, previous history of alcohol or sedative overdose, previous history of adverse events associated with propofol, sleep apnea or recent history of central nervous system (CNS) abnormalities (e.g. stroke) were excluded. Also, patients who refused sedation during colonoscopy, who were hospitalized or who were pregnant or lactating were excluded from the study.

The participants who were recruited, and who met the inclusion criteria, were consecutive individuals who underwent colonoscopy at the same unit, between January 30, 2017, and January 15, 2018. The fasting periods implemented were in accordance with ASA guidelines. All patients underwent colonoscopy preparation following the standard procedure of the endoscopy unit.

### Randomization and blinding

Patients were randomized, by using computer-generated block randomization (http://www.randomization.com), into two groups (1:1 allocation ratio). One anesthetist controlled the randomization table and another anesthesiology professional performed sedation, and this latter was blinded for allocation.

All sedation procedures were performed by an anesthesiologist (BŞ) who was blinded to the pre-procedure steps. Colonoscopy commenced when the anesthesiologist decided that the depth of sedation was adequate.

All endoscopy procedures were performed blindly in relation to this study, by one of three endoscopists, each of whom had performed more than 500 endoscopies before participating in this study.

### Interventions

In the monitored group (n = 50), the depth of anesthesia was monitored by means of the BIS (BIS Monitor, Aspect 2000 XP, USA) and BIS scores were maintained between 60-80. In the usual care group (n = 50), BIS monitoring was not performed.

The depth of sedation was calculated by measuring cerebral electric activity via an electroencephalogram (EEG). The BIS algorithm processed the frontal EEG and converted the signal to a waveform on the BIS monitor. The monitor calculated the data received by the two to four sensors and displayed this information as numerical values from 0 to 100 with a 10 to 30-second delay. Each numerical range correlated with a different degree of sedation: 100 to 90, awake and responding appropriately to verbal stimulation; 80 to 70, light to moderate sedation; 70 to 60, deep sedation; 60 to 40, general anesthesia; less than 40, deep hypnotic state; less than 20, burst suppression; and 0, totally suppressed EEG (flat line).^10^ The BIS measurement times in the present study were as follows: t_0: at baseline; t_1: immediately after induction; t_2: at the beginning of colonoscopy; t_3: at the 5^th^ minute of colonoscopy; and t_4: at the end of colonoscopy.

A 20-gauge intravenous catheter was inserted in the right forearm when the patient arrived in the endoscopy room. Supplemental oxygen (4 l/min) was administered through a nasal cannula. In addition to routine monitoring (consisting of use of a pulse oximeter, three-lead electrocardiogram (ECG) and non-invasive blood pressure cuff), BIS monitoring (BIS Monitor, Aspect 2000 XP, USA) was applied to the patients in the monitored group. After baseline measurements (hemodynamic profiles and BIS values) had been obtained, the patient was placed in the left lateral position.

Two milligrams of midazolam were administered intravenously. Next, an initial intravenous dose of propofol (0.3-0.5 mg/kg of body weight) was administered, followed by repeated 10-20 mg doses. In the monitored group, this was done until BIS values of 60-80 were reached or the patient expressed discomfort. In the usual care group, this was done until the patient’s sedation level was more than a score of 4 on the Modified Observer’s Assessment of Alertness/Sedation scale (MOAA/S)^22^ or the patient expressed discomfort. No other medications, including analgesics, were used in the present study.

### Outcomes

To determine the baseline levels of the patients’ cognitive performance, the MMSE,^23^ TDT^24^ and clock drawing test (CDT)^25^ were performed on the day of the procedure, after admission to the endoscopy unit. The MMSE evaluates orientation, registration, attention, calculation, recall, language and praxis (cutoff < 23, for abnormal).^23^ This test was used to quantitatively assess psychomotor activity.^24^ The TDT score represents the total number of dots that are not connected, with a total score of 70 points. The TDT scores and TDT deviations were normalized to baseline scores and deviations for each patient. The CDT is a simple neuropsychometric instrument that can easily be applied to assess several neuropsychiatric functions (scores from 0 to 5; cutoff: 3 points).^25^

Age, gender, ASA physical status, body mass index (BMI), total propofol dose, duration of sedation, patient satisfaction and MOAA/S on arrival at the post-anesthesia care unit (PACU) were recorded. Furthermore, heart rate (HR), mean blood pressure (MBP), oxygen saturation (SpO_2_) and BIS values (at baseline, immediately after induction, at the beginning of colonoscopy, at the 5^th^ minute of colonoscopy, at the end of colonoscopy and at discharge from the post-anesthesia care unit) were recorded. We also recorded any complications associated with sedation (i.e. oxygen saturation < 90%, blood pressure < 90/50 mmHg or HR < 50 bpm).

The patients were classified as ready for hospital discharge in accordance with the Chung criteria.^26^ Within these criteria, they were considered fit for discharge home when their score was ≥ 9 out of a total of 10. At this point, the patients’ satisfaction was evaluated (as dissatisfied, neutral or satisfied). In addition, post-procedural cognitive performance was assessed using the MMSE, TDT and CDT. The baseline and the post-procedural cognitive performance were assessed by another anesthesiologist (MSU), who was blinded to the BIS that had been used.

### Sample size

The sample size calculation was based on a prior pilot study with 16 patients (unpublished data). The primary outcome variable was the mini-mental state examination test score at discharge. The mean and standard deviation (SD) of the discharge MMSE test scores of the two groups were taken to be 25.30 (SD 3.83) and 23.30 (SD 2.98), as determined based on the preliminary study on 16 patients. It was calculated that, for a 25% difference between the groups, with a significance level of 0.05 and a power of 80%, 48 subjects would be required. Thus, 50 subjects were included, to cope with possible drop-outs.

### Statistical analysis

The statistical analyses were performed using the Statistical Package for the Social Sciences 15.0 software (SPSS Institute, Chicago, IL, USA). Continuous data were tested for normality. Normally distributed data were summarized using the mean and standard deviation and were compared using unpaired two-tailed t tests. Skewed data were summarized using the median with the range and interquartile range (IQR) and were compared using Wilcoxon’s rank sum test. Categorical data were summarized using the number and percentage (%) and were compared using the chi-square (X^2^) test or Fisher’s exact test. Paired data were compared using paired two-tailed t tests or signed-rank tests. P-values less than 0.05 were considered statistically significant.

## RESULTS

**Figure 1.**
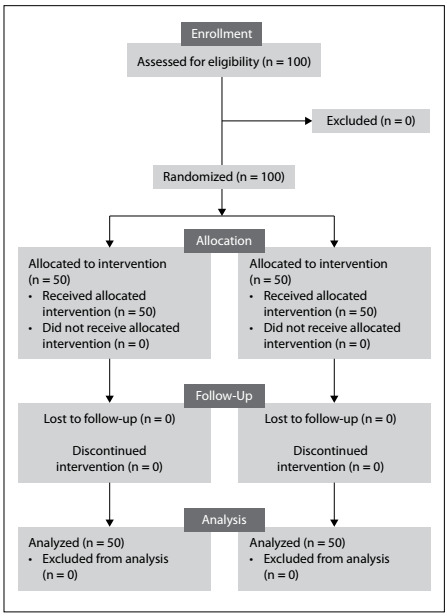
CONSORT flowchart detailing patient recruitment.

A total of 100 patients were enrolled in the study, and all patients completed the investigation. Figure 1 shows the Consolidated Standards Of Reporting Trials (CONSORT) flow chart detailing patient recruitment. Data analysis was performed on the two groups. No patient was withdrawn from the study after induction of sedation, and no complication developed.

The patients’ demographic data are summarized in Table 1. There were no significant differences between the groups regarding age, gender, ASA physical status or BMI.

**Table 1.** Patients’ characteristics

	Monitored group (n = 50)	Usual care group (n = 50)	P
Age (years), mean (SD)	47 (13)	48 (14)	0.722*
Gender (male/female)	31/19	28 / 22	0.462^#^
ASA physical status, number (%)			
I	29 (58)	30 (60)	0.763^#^
II	21 (42)	20 (40)	
BMI (kg/m^2^), median (range) [IQR]	27 (16-36) [24-31]	26 (15-36) [21-29]	0.143^$^

*Unpaired two-tailed t tests; ^#^χ^2^ test; ^$^Wilcoxon’s rank sum test.

SD = standard deviation; ASA = American Society of Anesthesiologists; BMI = body mass index; IQR = interquartile range.

**Figure 2.**
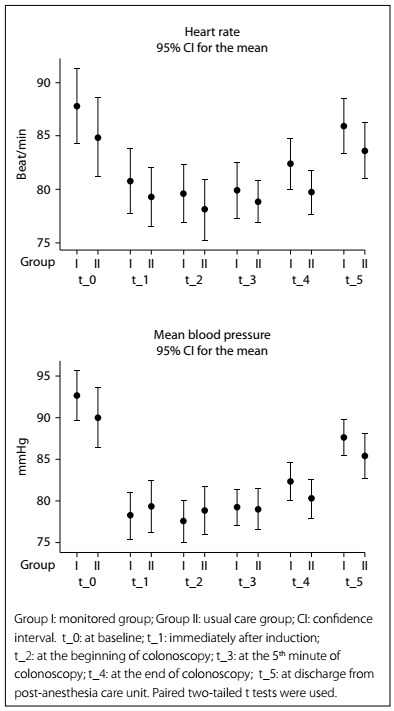
Changes to heart rate and mean blood pressure.

The changes in HR and MBP can be seen in Figure 2. The measurements of hemodynamic parameters (HR and MBP) were statistically similar between the groups at all measurement times.

Sedation, procedure and recovery characteristics are summarized in Table 2. There were no significant differences in the duration of sedation or in the patients’ satisfaction between the groups. The total amount of propofol used in the sedation procedure was statistically significantly lower in the monitored group than in the usual care group (P < 0.001).

**Table 2.** Sedation, procedure and recovery characteristics

	Monitored group (n = 50)	Usual care group (n = 50)	P
Total propofol dose (mg), median (range) [IQR]	100 (50-200) [100-140]	150 (75-200) [100-200]	< 0.001^$^
Duration of sedation (min), median (range) [IQR]	16 (10-25) [15-20]	16 (11-22) [12-20]	0.197^$^
MOAA/S on arrival at PACU, median (range) [IQR]	3 (2-4) [3-4]	4 (2-4) [3-4]	0.001^$^
Patients’ satisfaction, number (%)			
Dissatisfied	3 (6)	5 (10)	0.260^#^
Neutral	10 (20)	10 (20)	
Satisfied	37 (74)	35 (70)	

IQR = interquartile range; MOAA/S = Modified Observer’s Assessment of Alertness/Sedation scale; PACU = post-anesthesia care unit. #: χ^2^ test, $: Wilcoxon’s rank sum test.

The changes in BIS values in the monitored group BIS are summarized in Table 3.

**Table 3.** Changes to bispectral index (BIS) values in monitored group

Time	BIS values*
t_0	97 (92-98) [96-98]
t_1	66 (55-78) [61-72]
t_2	65 (58-77) [62-70]
t_3	71 (59-80) [68-74]
t_4	78 (68-86) [75-81]

*Values are expressed as median (range) [interquartile range, IQR]. t_0: at baseline; t_1: immediately after induction; t_2: at the beginning of colonoscopy; t_3: at the 5^th^ minute of colonoscopy; t_4: at the end of colonoscopy.

The patients’ cognitive function test results at baseline and discharge are summarized in Table 4. There were no significant differences between the groups regarding the baseline values of the cognitive function test results (MMSE, TDT and CDT). The discharge values of the MMSE and CDT were statistically significantly higher in the monitored group than in the usual care group (P = 0.002 and P = 0.002, respectively). Moreover, the discharge values of the TDT were statistically significantly lower in the monitored group than in the usual care group (P = 0.033).

**Table 4.** Cognitive performance tests on the patients at baseline and discharge

	Monitored group (n = 50)	Usual care group (n = 50)	P*
MMSE, mean (SD)			
Baseline	27 (3)	26 (2)	0.113
Discharge	26 (3)	23 (3)	0.002
TDT, mean (SD)			
Baseline	8 (6)	7 (5)	0.057
Discharge	11 (7)	15 (11)	0.033
CDT, mean (SD)			
Baseline	3 (1)	3(1)	0.584
Discharge	3 (1)	2 (1)	0.002

*Unpaired two-tailed t tests. MMSE = mini-mental state examination test; SD = standard deviation; TDT = Trieger dot test; CDT = clock drawing test.

## DISCUSSION

The current study demonstrated that BIS monitoring of patients subjected to sedation for colonoscopy was associated with diminished use of propofol and better scores in post-procedure cognitive performance tests than what was observed among patients who were not monitored.

Postoperative cognitive function disorder is a common neurological complication seen after surgery, anesthesia and sedation. It is characterized by impairments in recent memory, concentration, language comprehension and social integration.^27^ The effect of anesthetic depth on postoperative cognitive dysfunction among surgical patients has already been investigated using BIS monitoring,^17^ and various medications have been compared in studies assessing POCD subsequent to procedures such as colonoscopy and ERCP.^1^^,^^18^ However, no previous study has evaluated the effects of BIS monitoring on early cognitive performance after outpatient procedures such as colonoscopy and ERCP.

To reduce the amount of sedative agents administered to patients, sedation depth can be monitored using objective and reliable methods such as BIS monitoring. BIS, obtained through use of a conventional electroencephalogram, is independent of the type of hypnotic medications and individual patient characteristics. However, some agents such as ketamine and opioids are not suitable for BIS monitoring.^11^ Previous publications on the use of BIS for monitoring sedation during colonoscopy mostly focused on decreasing the amount of sedative agents administered, or on assessing recovery time or patients’ or endoscopists’ satisfaction.^12^^,^^13^^,^^14^^,^^15^^,^^16^

It has been demonstrated that intraoperative BIS monitoring facilitates the titration of anesthetic agents. Thus, it has the potential to decrease the use of anesthetic agents.^28^^,^^29^^,^^30^ BIS monitoring has also been shown to enable improvement in the early recovery profile through reducing the use of anesthetic agents.^19^^,^^20^ However, it is unclear whether the reduced dose of anesthetic agents achieved through the use of BIS monitoring decreases the risk of POCD.

In sedated patients, BIS monitoring has been shown to result in decreased propofol use, shortening of the duration of awakening and reduction in the number of adverse events.^21^ The decrease in the amount of sedative agents used during colonoscopy procedures that were accompanied with BIS monitoring, as previously reported in the literature, were confirmed through the present study as well. In the present study, the amount of propofol used during the procedure was significantly lower in the group that underwent BIS monitoring than in the group without BIS intervention. Post-procedure cognitive performance was significantly better in the monitored group, possibly due to reduced use of sedative agents.

Although it has been reported in several studies that BIS monitoring reduced the use of propofol in sedated patients, contrary results have also been demonstrated in some other studies.^16^^,^^31^ Unlike in our study, Imagawa et al.^13^ reported that BIS monitoring did not reduce the use of propofol during sedation, but that it improved patient satisfaction scores. Similar to our findings, Yu et al. did not find any effect from BIS monitoring on patient satisfaction scores.^16^

Although studies have evaluated the effects of general anesthesia accompanied by BIS monitoring on cognitive dysfunction, no previous study has investigated early cognitive performance among patients sedated under the guidance of BIS monitoring.^32^^,^^33^ In a previous study that included geriatric patients, use of intraoperative BIS monitoring was associated with decreased incidence of postoperative delirium, but no decrease in the rate of POCD was noted.^32^ Another study among geriatric patients demonstrated that intraoperative use of BIS monitoring decreased the rates of both postoperative delirium and cognitive dysfunction.^33^

In this study, the period between the termination of the procedure and the second application of cognitive tests was not recorded, and this was one limitation of the study. The second limitation of this study was the absence of longer follow-up to assess cognition several days after the procedure.

## CONCLUSION

The results from our study suggest that BIS monitoring during colonoscopy in sedated patients gives rise to in a decrease in the amount of propofol used during the procedure and that it possibly precludes cognitive performance decline. In addition, the lower MOAA/S values on arrival at the PACU that were detected in the group sedated under the guidance of BIS monitoring can be considered to be a consequence of the decrease in the propofol dose used during the procedure.
